# High expectations: Civil society participation in conflict early warning and response systems of the AU, ECOWAS and IGAD

**DOI:** 10.1080/10220461.2024.2385935

**Published:** 2024-08-21

**Authors:** Michael Aeby

**Affiliations:** a University of Basel, Basel, Switzerland; b University of Cape Town, Cape Town, South Africa

**Keywords:** Conflict early warning and response systems, civil society participation, AU, ECOWAS, IGAD

## Abstract

Civil society organisation (CSO) participation in conflict early warning and response systems (CEWRS) of African intergovernmental organisations is expected to be beneficial to collect data, conduct analyses, and respond timeously to the potential escalation of violent conflict in a way that is relevant to local stakeholders. These high expectations contrast with CEWRS’ protracted operationalisation procedures, low capacity of many CSOs, and the challenges of integrating civil society into (inter)governmental structures, which militate against effective participation. This article considers the net effect of CSO participation, examining how CSOs contributed to data collection, analyses, early warning and responses of CEWRS in the AU, ECOWAS and IGAD, and assesses their participation models. The article finds that CSO participation is a long way from delivering on expectations. It argues that CSOs can only fully deliver by building independent CEWRS that complete early warning-response processes in parallel to the intergovernmental systems, as in West Africa.

## Introduction

The Intergovernmental organisations (IGOs) that constitute building blocks of the African Peace and Security Architecture (APSA) have partnered with civil society organisations (CSOs) to bolster the capacity of their conflict early warning and response systems (CEWRS), as well as render their prevention activities more inclusive.^1^ Contributing to literature on conflict prevention through the APSA, this article examines whether civil society participation has delivered expected benefits for CEWRS in the African Union (AU), the Economic Community of West African States (ECOWAS) and the Intergovernmental Authority on Development (IGAD), and assesses comparative advantages of the participation models in each of these cases. This introduction will outline the literature on conflict early warning systems worldwide, African CEWRS, and civil society in conflict prevention that provides the conceptual framework. Furthermore, it explains the state of research on civil society participation in the CEWRS of the APSA, research design, and key findings. The article will proceed to present three case studies on the AU, ECOWAS and IGAD, which examine policy frameworks, the operationalisation of CEWRS, and the involvement of CSOs in data collection, analysis, warning and responses. The article will then juxtapose the participation models in the three IGOs to draw conclusions on their relative advantages.

### Conflict early warning and response systems

In the 1990s, mass atrocities prompted IGOs, governments and non-governmental organisations (NGOs) to develop conflict ‘early warning’ and ‘early response’ systems to anticipate, avert and mitigate a range of potential violent conflicts. ‘Early warning’ describes a process that alerts decision-makers and promotes their understanding of the nature and impact of a potential escalation of violent conflict. Early warning systems, as a subset of CEWRS, constitute the procedures to provide regular forecasts and predictive capacity for proactive evidence-based decision-making, programming and priority-setting.^2^ While early warning systems inform decision-makers, other types of CEWRS also effectuate ‘early responses’ – ie, actions that are informed by early warning and timely deployed to prevent or mitigate violence.^3^ CEWRS have evolved into a variety of models. These include centralised early warning systems to inform political elite actors; decentralised systems that harness community-level information and responses; quantitatively-oriented systems that leverage socio-economic, (social) media and geo-data; and systems that use artificial intelligence to generate situation analyses and decision-options in real-time.^4^

The elementary processual steps of all CEWRS entail data collection, analysis, warning and response. However, CEWRS vary in their security doctrines, conflict types, data sources, analytical methods, recipients of warnings, and responders. In contrast to intelligence organisations, CEWRS mainly rely on open-source information from news media, field monitors and government agencies.^5^ Quantitative and qualitative data on events and structural vulnerabilities is processed through algorithmic forecasting models and interpretative conflict analyses. New technologies are hoped to lead to better forecasting.^6^ Yet doubts persist whether complex idiosyncratic conflict dynamics that result from human actions can be reliably predicted through reductive models.^7^

High-level political decision-makers are the main recipients of confidential warning reports in (inter)governmental CEWRS. Warnings may inform responses including preventive diplomacy, mediation and peacekeeping in acute crises, or long-term prevention through policymaking to address structural vulnerabilities. In decentralised systems that register grassroots conflicts, responders may include local-level state and non-state actors.^8^ The persistent early warning-response gap – ie, the challenge of translating early warning information into preventive action – is regarded as CEWRS’ foremost limitation. The gap is often blamed on the inadequacy, unreliability and poor communication of early warning reports, and on decision-makers’ lack of ‘political will’ to take costly preventive action before conflict escalates.^9^

### CEWRS in the APSA

The AU’s predecessor, the Organisation for African Unity, along with various African regional economic communities (RECs), built conflict prevention mechanisms even before the 2002 Protocol of the AU Peace and Security Council (PSC), which set out the structures of the APSA.^10^ The protocol promoted collaboration between the AU Continental Early Warning System (CEWS) and the RECs’ individual conflict early warning and response systems.^11^ The African early warning systems were designed to inform IGOs’ high-level decision-makers holding the authority to activate IGOs’ separate response mechanisms, as well as decentralised CEWRS with built-in national response functions in member states. Besides the AU, ECOWAS and IGAD, the Common Market for East and Southern Africa (COMESA), the Community of Central African States (ECCAS), the East African Community (EAC) and the Southern African Development Community (SADC) operationalised CEWRS.^12^ The systems prioritised different conflict types and indicators but converged technologically in that the AU, COMESA, ECOWAS and ECCAS used software developed for IGAD’s reporter network.^13^ Close observers note, however, that whereas AU and REC early warning officers held regular coordination meetings, competition and divergent security doctrines have complicated collaboration and information-sharing.^14^

Although the capacity of the African CEWRS has reportedly improved, their ability to prevent conflict has continued to be a matter of scepticism due to tedious operationalisation processes, exacerbated by a lack of resources, technical expertise and prioritisation by decision-makers.^15^ Researchers highlighted the warning-response gap. The warning-response gap, they explained, was due to a mismatch between the reports of the low-ranking early warning officers and the expectations of the high-level decision-makers, who needed tailor-made actionable recommendations rather than generic conflict analyses. Decision-makers could access classified intelligence and diplomatic sources they deemed superior to the reports from the CEWRS, often based on open-source information.^16^ African CEWRS were therefore underutilised because member states, which were often conflict actors themselves and sometimes seeking to avoid scrutiny for human rights abuses, resisted interventions in intrastate conflicts on the basis that such intervention infringed on their sovereignty.^17^

### Civil society participation in CEWRS

Various works within the literature on local peacebuilding and inclusive peace-making credit civil society with a vital role in preventing and transforming conflict.^18^ Civil society is here understood as ‘a sphere of uncoerced association between the individual and the state, in which people undertake collective action for normative and substantive purposes, relatively independent of government and the market’.^19^ As such, the term civil society encompasses various actors who may promote peaceful societal relations – or instigate violent conflict.^20^ African CSOs that are relevant to CEWRS and this study include (a) local-level community-based and national-level organisations in conflict-affected countries, (b) highly professionalised expert peacebuilding NGOs and research institutions, and (c) regional networks that are coordinators and intermediaries between CSOs and IGOs. Empirically, the distinction between these CSO types and the boundaries between civil society, economic role players and state actors are not clear-cut.

Thanks to their proximity to communities, local CSOs are considered well-placed to register and prevent grassroots conflict through responses that are accepted and relevant to communities.^21^ Local actors may gather early warning information as field monitors and through cell phone-based crowdsourcing platforms, while community leaders respond to low-intensity conflict by promoting dialogue.^22^ Expert NGOs and research institutions are deemed better suited for independent analyses than state actors, whose judgment may be clouded by governments’ interests and involvement in conflicts.^23^ Regional networks may coordinate civil-society-based CEWRS or channel information towards (inter)governmental systems.^24^ Participation in CEWRS must, moreover, be seen in the context of the inclusive peace paradigm, the incorporation of ‘inclusivity’ as a norm in African IGOs’ policies, and IGO’s strategies to legitimise their role in managing conflict.^25^ In sum, thanks to their proximity to communities, local knowledge, expertise and independence, CSOs are deemed in the peacebuilding literature to be well-suited to contribute to data collection, analyses, warning and responses. Participation by CSOs in IGO prevention activities is hoped to augment the responsiveness and legitimacy of those activities vis-à-vis civic stakeholders.

Research on the CEWRS of the AU, ECOWAS and IGAD between 2002 and 2021, cited below, offers valuable insights on CSO participation at earlier stages. The warning systems have undergone extensive reforms in recent years, however, altering CSO involvement. The changes, firstly, included the AU’s promotion of sub-regional NGO networks to feed into the AU CEWS.^26^ Secondly, IGAD’s Conflict Early Warning and Response Network (CEWARN), which focused on pastoralist conflicts in cross-border clusters, was transformed to monitor a comprehensive range of potential conflicts across member states.^27^ Thirdly, the ECOWAS Conflict Warning and Response Network (ECOWARN) introduced national centres.^28^ Fourthly, the IGOs introduced and elaborated response mechanisms for preventive diplomacy and mediation.^29^ These far-reaching changes to warning and response processes warrant a reassessment of CSO involvement. This article makes a valuable empirical contribution to the literature on conflict prevention under the auspices of the APSA by analysing how CSOs participated in early warning and response processes in three IGOs between 2021 and 2023. It provides a critical assessment of whether CSOs were able to deliver expected benefits, among other contributions, by enriching data, adding alternative views, and enabling swift responses.

### Research design

The study primarily aims to assess whether civil society participation in the early warning and response processes of the AU, ECOWAS and IGAD has delivered benefits that were expected in early warning literature and IGO policies. Secondly, it serves to identify comparative advantages of the participation models of the various IGOs under scrutiny. In particular, it explores how CSOs participated in the data collection, analytical processes and responses in practice, and assesses whether CSOs could meaningfully improve CEWRS’ data collection, analyses and responsiveness. More specifically, it seeks to ascertain whether CSOs are able to enrich data collection with information on local-level dynamics, add alternative perspectives to analyses and warnings, and partake in preventive and responsive action. Rather than focusing solely on dedicated CEWRS structures, it considers IGOs’ wider warning-response processes, how IGO policies envisioned participation, and how CSOs contributed – or failed to contribute – to CEWRS’ operationalisation.

The article explores these questions in case studies on the AU, ECOWAS and IGAD, which were selected because their CEWRS involved CSOs through different models and were among the APSA’s most developed systems.^30^ A case study on the Southern African Development Community (SADC) was excluded after an assessment turned up no noteworthy civil society participation in the Regional Early Warning Centre.^31^ The conclusion juxtaposes the systems’ participation models to highlight advantages.

The analysis is based on 60 semi-structured key-informant interviews with representatives of IGOs, CSOs and research institutions, who were selected owing to their involvement in CEWRS in a non-representative snowball sample. It builds on an extensive review of policies and literature that cannot be detailed. The perspectives of the interviewees, 24 of whom chose to remain anonymous, heavily inform the exploratory study’s findings. Since most interviews were done between 2021 and 2023, the study reflects the systems in this period.

### Key findings and argument

The three cases show that participation could, in principle, improve data collection, analyses and responses, but, in practice, hardly lived up to the above detailed expectations: Outside West Africa and in insecure and authoritarian settings, NGO networks lacked local monitors, capacity and civic freedom to meaningfully complement and evaluate media-based data.^32^ In all sub-regions, IGO and government officials, who committed to participation in policies but remained suspicious of non-state actor involvement in managing security, provided little room for CSOs to substantively inform analyses, warnings and responses.^33^ Eastern and Southern African NGOs that fed information into the AU and IGAD systems were denied access to aggregated data and analytical outputs.^34^ Thanks to its free-standing CEWRS, the West Africa Network for Peacebuilding (WANEP), in contrast, could not only feed information into the AU and ECOWAS system, but conduct independent analyses to warn civic stakeholders, governments and ECOWAS with alternative findings, and devise local responses by WANEP affiliates.^35^

Based on these findings, the study argues that participation can fully deliver only if regional blocks emulate the ECOWAS-WANEP model, which requires NGO networks to establish independent civil society-based CEWRS and link them to IGOs rather than solely feed selective information into (inter)governmental systems at individual stages. Independent civil society CEWRS, which complete the basic steps of the early warning-response process in parallel to IGOs, are essential to achieve so that CSOs can meaningfully complement data with information on human security concerns that state actors may ignore, enrich analysis with deviant perspectives based on independent data, and enable timely local responses.

## The AU continental early warning system

Established as an APSA pillar under the PSC Protocol of 2002, the AU CEWS became operational in 2012. It was created to monitor matters relating to politics and governance, social and economic affairs, extremism, crime, environment and gender, all of which were considered potential points of contention that might lead to violent conflict. Its structures comprised a situation room and analysts in the early warning division. The early warning division had the narrow mandate of briefing the AU Chair. The latter alerted the PSC to decide on AU responses, deployed special envoys or the Panel of the Wise to undertake preventive diplomacy and mediation, and informed AUC departments, RECs and governments.^36^ Following the 2021 reforms of the AU Commission (AUC), although mainly serving conflict prevention, the division was integrated into the Conflict Management Directorate of the merged Department of Political Affairs, Peace and Security (PAPS). According to close observers, the AUC reform disrupted the CEWS procedures, with far-reaching implications for the participation of CSOs, and the system’s future hung in the balance at the time of writing.^37^

### AU policy framework for CSO participation

**Figure 1.**
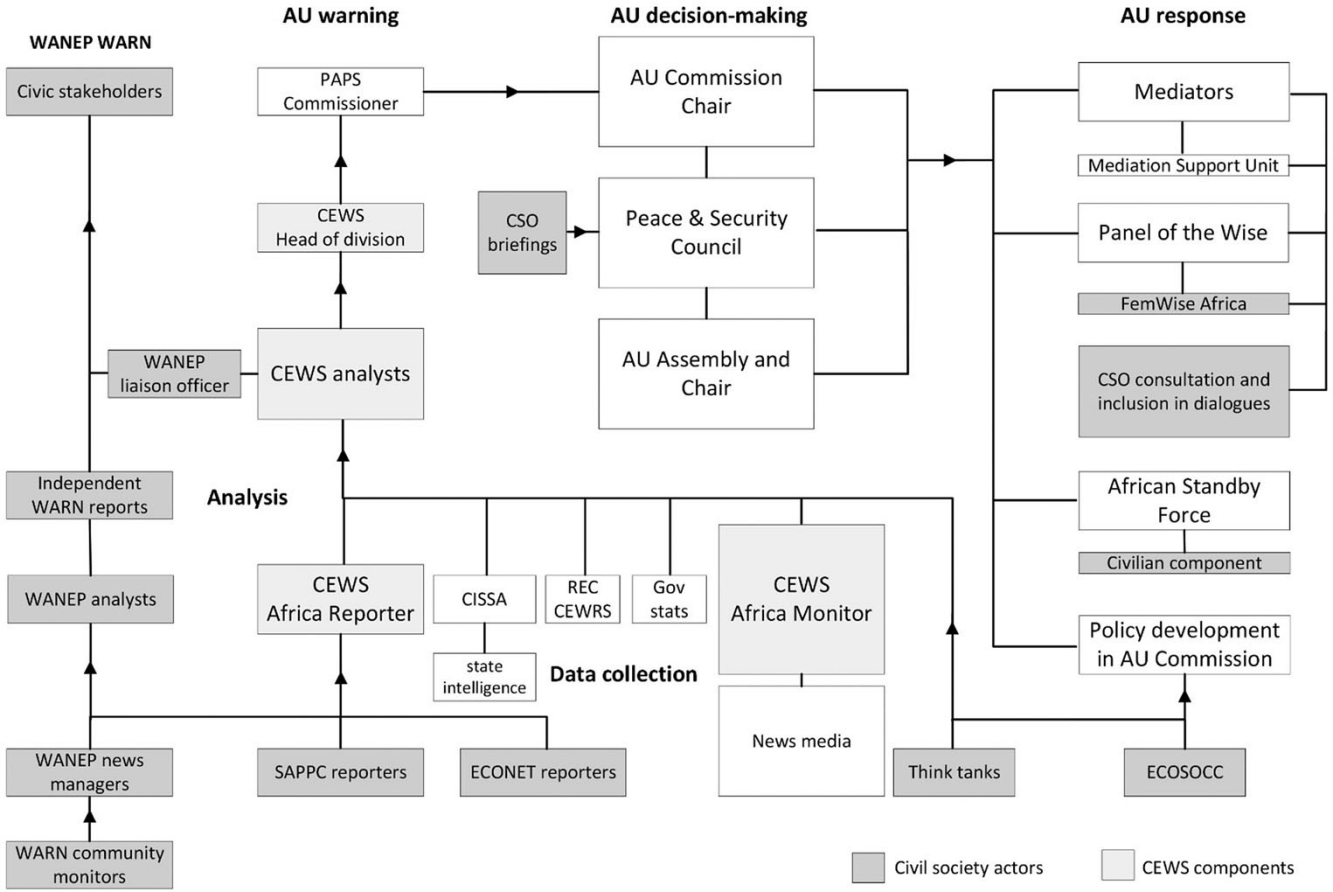
CSOs in AU early-warning-response processes. *Sources:* The author created this organigram based on the interviews and documents cited in the AU case study.

An array of AU policies envisioned participation at all stages of the warning-response process (see Figure 1). Guidelines for the CEWS underscored that early warning should be done in a ‘participatory manner’ to enable ‘empowered citizenship’, identifying civil society actors as sources of information, experts for analyses, and local responders.^38^ CSOs should brief the PSC to inform decision-making, and expert NGOs should advise envoys, who should consult civic stakeholders in preventive and mediation missions. The AU prioritised those CSOs that were represented in the Economic, Social and Cultural Council (ECOSOCC), which restricted donor-funding for its members organisations. The PSC, however, introduced the principles of relevance and flexibility to permit collaborations with donor-reliant expert NGOs.^39^ As AU policies contained restrictions, grey areas and little detail on participation modalities, however, such partnerships stood on shaky ground.^40^

### AU CEWS operationalisation

The AU CEWS was operationalised between 2003 and 2012, receiving technical support from the Institute for Security Studies (ISS), ACCORD and the Kofi Anan International Peacebuilding Training Centre (KAIPTC), which assisted with the conceptualisation of its structures and offered training.^41^ The involvement of AU-external actors also sparked criticism among scholars: whereas Jackie Cilliers found consultants promoted inept commercial technologies,^42^ Ulf Engel argued that experts conveyed knowledge rooted in Western epistemological traditions.^43^ After the launch of the AU CEWS, involvement of these actors was limited to consultative workshops and sporadic analytical inputs.^44^ In general, non-state actor participation, although enshrined in AU policies, lacked unequivocal member-state support.^45^ Since governments viewed various NGOs with suspicion, the AU CEWS instead partnered with regional networks that were perceived more positively.^46^

### AU CEWS data collection

The AU CEWS relied on news agencies, socioeconomic statistics and, to a lesser extent, field monitors and think tank reports for its data. It received analytical reports from CEWRS of RECs and liaised with the AU Committee of Intelligence and Security Services. In 2010, the Africa Media Monitor became the principal collection instrument. The automated text search system was developed with the European Union's Joint Research Centre to extract data from news wire services.^47^ In 2013, the AU CEWS introduced the Africa Reporter to gather information that the popular media may not cover on grassroots-level developments, employing field reporters who would periodically issue situation and incident reports on 73 analytic domain indicators relating to the AU CEWS. The staff of AU liaison offices initially served as reporters. However, a high turnover and workload among reporters resulted in inconsistent reporting and rendered the model unworkable; in 2015, the CEWS thus followed its counterparts in subregions, CEWARN and ECOWARN, by turning to civil society to sustain the Africa Reporter. In doing so, it harnessed existing and promoted new NGO networks.^48^

In West Africa, the AUC entered a memorandum of understanding (MoU) with ECOWAS’ partner, the West Africa Network for Peacebuilding (WANEP). WANEP’s news managers in 15 countries, henceforth, answered questionnaires for the AU CEWS and the ECOWARN Reporter, using data from community monitors in WANEP’s independent Warning and Response Network (WARN). To encourage NGOs in other sub-regions to emulate WANEP’s structures and partnership, the AUC organised knowledge exchange events. Analogous to WANEP, sub-regional networks were intended to raise funding from donors using the AU’s credentials.^49^

To recruit reporters in the SADC region, the AUC chose the Southern Africa Partnerships for the Prevention of Conflict (SAPPC), a loose network of peacebuilding NGOs affiliated with the Global Partnership for the Prevention of Conflict (GPPAC). By 2021, SAPCC country managers in Botswana, Lesotho, eSwathini, Mozambique, Tanzania, Zambia, and Zimbabwe fed into the Africa Reporter, drawing from 40 community monitors in local CSOs. It must be noted, however that SAPPC lacked reporters in nine SADC countries, including South Africa, where the affiliate, ACCORD, did not participate in the GPPAC-initiated programme.^50^

To cover the IGAD and EAC regions, the AUC helped launching the Eastern Africa Civil Society Network (ECONET), which by 2021 comprised 90 NGOs and featured country reporters in Kenya, Tanzania, Uganda and South Sudan but lacked a secretariat. As ECONET, as of the time of writing, was yet to establish national chapters and networks of community monitors, country reporters relied on individual informers.^51^ In 2023, a donor-sponsored effort to capacitate and integrate an ECCAS partner, the Coalition of CSOs for Peace and Conflict Prevention in Central Africa (COPAC), made little headway as the network fell short of basic requirements and lacked reporters.^52^

According to representatives of the AU CEWS and of WANEP, the reporter network significantly improved the data, as it registered local-level dynamics in 15 West African countries, dynamics which otherwise went unheeded in media coverage.^53^ As the Central, Eastern and Southern African networks were in their infancy and lacked community monitors, their contribution was minuscule. Interviewed network reporters believed that they added value to monitoring efforts through their knowledge of vernacular languages and community concerns, and by giving human security matters prominence in the data. They also noted the need to counter media biases and recognise state propaganda. Yet, SAPPC and ECONET country reporters in seven states suggested that without community monitors, reporters depended on news and government sources and struggled to discern disinformation.^54^ The added value was, thus, largely reduced to the interpretative selection of information from a civil society perspective.

Since ECONET and SAPCC could not offer compensation and relied on volunteers, a high turnover of reporters and community monitors eroded training and networking efforts. Although assisting the AU, reporters often were forced to work clandestinely to evade suspicion by security agencies in various stages. Reporters in several East and Southern African countries highlighted that peacebuilding NGOs were tightly regulated, and community members were reluctant to brief reporters, fearing criminals, extremists and security agencies.^55^

Overall, participation in data collection was, thus, still a long way from delivering the expected benefits of complementing and verifying media-based information on a meaningful scale. Emulating the system WANEP built over 20 years to feed into ECOWARN and the AU CEWS in other sub-regions would hinge on NGOs’ ability to maintain steady donor relations and sturdy structures, and states’ willingness to provide sufficient civic space.

### AU CEWS analysis

Prior to the AUC reform, automated systems and early warning officers at the CEWS’ situation room processed all data for analyses: The Africa Media Monitor automatically generated the African Brief news page that highlighted relevant developments every 10  minutes and visualised locations of events on a live monitor. A news desk permitted analysts to select information requiring the attention of decision-makers. The Africa Prospects programme assessed structural vulnerabilities of countries based on statistical regression.^56^ The Africa Reporter, whose software was designed for CEWARN, identified predictors for conflict escalation based on situation and incident reports.^57^ Five regional analysts assessed political, security, economic and cultural factors using techniques including timelines, conflict-mapping and scenario-building.^58^

In the AUC reform, the analysts were redeployed to regional desks and charged with conflict management rather than early warning. The situation room was retained, but sources suggest the CEWS was paralysed as analysts could no longer execute standard operations such as issuing narrative reports and horizon scans for the PSC. AU-REC coordination meetings and country vulnerability analyses were suspended. Whereas observers differed on the severity of the disruption, there was a general consensus that the expertise and instruments built for the CEWS were at risk. Although the PSC Protocol stipulated the mandate of the CEWS, the latter’s functions and procedures became subject to contestations in the PAPS and PSC.^59^

NGOs were involved in analyses through inputs by think tanks and an embedded WANEP liaison officer. The CEWS occasionally leveraged independent NGO reports and requested think tanks to provide specific analytical inputs. WANEP contributed country reports, data and expertise on demand, for instance for Ghana’s vulnerability assessment.^60^ Given the CEWS’s thematic range and staff turnover, think tank representatives struggled to identify focal points for such analytical inputs and questioned whether they were, indeed, utilised.^61^

WANEP’s liaison officer participated in analyses on West Africa, but the AUC reform likewise disrupted the civil society analyst’s integration. Successive WANEP liaison officers saw their added value in independent analyses and ‘outside the box’ recommendations. However, diplomatically phrased reports to decision-makers left minimal room for alternative findings and response options that might incriminate states or threaten to infringe their sovereignty. NGO contributions to analytical reporting were, thus, limited. Still, the partnerships and the embedded WANEP officer must be viewed as remarkable achievements, hopefully contributing to changed attitudes vis-à-vis civil society participation in PAPS.^62^ In this vein, the launch of the African Network of Think Tanks for Peace by the AUC in 2023 appeared to provide a chance to draw in research institutions. However, at the time of writing, the network had not been formally integrated into early warning analyses of the CEWS.^63^

### AU CEWS warning and response

The AU CEWS was designed to exclusively alert decision-makers responsible for prompting AU response mechanisms. The PSC Protocol, in principle, restricted information-sharing to the vertical escalation of reports via the division and department leaders to the AUC Chair. In practice, these office bearers received warnings simultaneously via text message, and the AU CEWS shared information horizontally with response mechanisms including the Mediation Support Unit (MSU) to inform mediation missions. Early warning information was, however, not systematically harnessed for policymaking across departments.^64^

On the basis of this study, it is clear that the communication of AU CEWS warnings did not involve non-state actors, though the PSC occasionally invited NGOs to submit written briefings and address the council in open sessions.^65^ These included international humanitarian organisations, human rights NGOs and local women’s groups. However, the periodic analytical briefings envisaged by PSC resolutions did not materialise. To inform PSC decisions, African think tanks systematically provided custom-made analyses to council members’ embassies, instead.^66^

Whereas AU response mechanisms were mainly designed to manage conflict on the elite-level rather than build peace on the local level, AU’s preventive diplomacy, mediation and peace-keeping missions involved NGOs and local civic stakeholders.^67^ The Panel of the Wise, whose mandate entailed preventive diplomacy and advising the PSC, produced studies with think tanks and consulted stakeholders on field missions.^68^ Its FemWise programme trained over 400 women mediators from CSOs for multitrack dialogues.^69^ The MSU used think tank reports to prepare mediation missions of special representatives, who routinely consulted civic leaders.^70^ The African Standby Force’s civilian component comprised non-governmental experts. To develop policy responses to structural vulnerabilities, the PAPS collaborated with think tanks. The prioritisation of ECOSOCC members over expert NGOs, however, hampered such collaborations.^71^

In sum, the AU’s early-warning-response processes offered CSOs minimal room to inform decision-makers, but NGOs used informal channels to sway PSC decisions. Inclusion in peace diplomacy was confined to consultations; this meant that think tanks’ potential to manage knowledge and develop policies was not fully harnessed.^72^

## The ECOWAS early warning and response network

**Figure 2.**
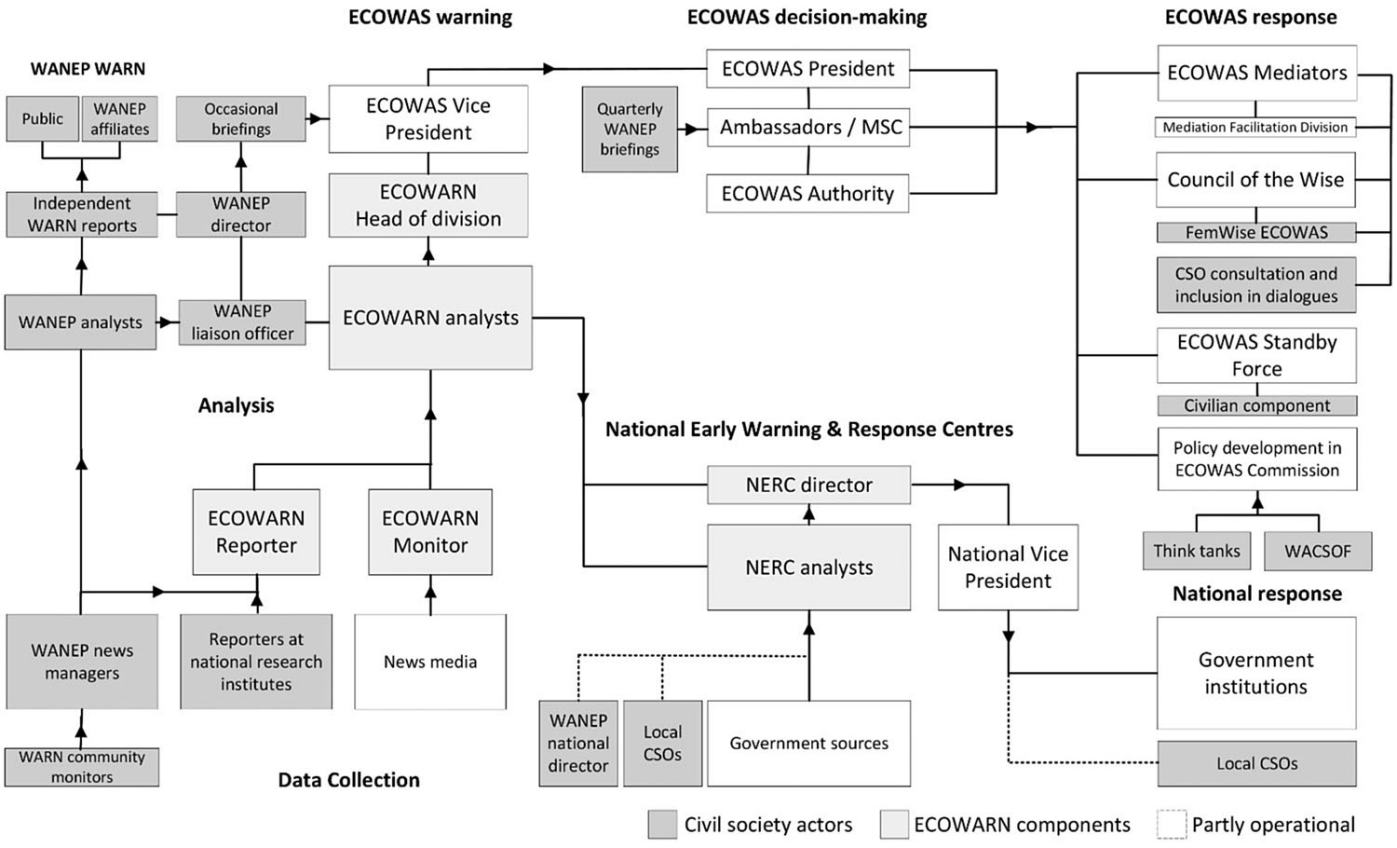
CSOs in ECOWAS early-warning-response process. *Source:* The author created this organigram based on the interviews and documents cited in the ECOWAS case study.

ECOWARN, established by the Protocol of the Mechanism for Conflict Prevention, Management, Resolution, Peacekeeping and Security of ECOWAS in 1999, went through several rounds of reform.^73^ It centred on human security and gathered information through 92 monitors in 15 ECOWAS countries focusing on five areas: politics and governance, economy, population, rule of law, and security.^74^ The central Early Warning Directorate featured a situation room and was located in the Office of the ECOWAS Commission’s Deputy President. The latter was the principal recipient of warnings, but could involve the ECOWAS president and ambassadors to devise responses. Whereas ECOWARN initially functioned as a centralised early warning system, in 2017 it was restructured to strengthen national response capabilities, transferring responsibility for responses from ECOWAS to member states. Zonal offices were replaced with National Early Warning and Response Centres (here NERCs) under the authority of member state deputy presidents. By 2023, Burkina Faso, Côte d’Ivoire, Ghana, Guinea-Bissau, Liberia and Mali had to varying degrees capacitated NERCs, while nine other member states had not launched theirs (Figure 2).^75^

### ECOWAS policy framework for conflict prevention

Policies including the ECOWAS Conflict Prevention Framework (ECPF) envisioned participation by CSO’s in all aspects of conflict prevention and transformation, to leverage local resources, promote ownership and human security and build an ‘ECOWAS of the People’^76^ The ECPF identified CSOs as partners for data collection, analysis, and local responses, as well as for training at the original zonal bureaus that would be replaced by NERCs.^77^ As inclusion constituted a mediation principle of ECOWAS, envoys and the Council of the Wise should engage civic stakeholders and encourage multitrack dialogues.^78^

### ECOWARN operationalisation

The operationalisation of ECOWARN involved WANEP from the onset. The network that launched in 1998 to coordinate peacebuilding NGOs made the provision of services to build ECOWARN as its mainstay and developed its independent WARN system in parallel. WANEP became ECOWARN’s main partner for training, prevention and mediation support after signing an MOU in 2004.^79^ ECOWARN and WANEP were hailed for fostering a culture of prevention, participation and human security.^80^ WANEP representatives advocated the proliferation of the partnership model to other CEWRS to enable grassroots participation.^81^ However, Amandine Gnanguênon found that CSOs had little stake in responses and cautioned against ECOWARN's dependence on a single service provider.^82^

### ECOWARN data collection

ECOWAS’ Early Warning Directorate collected data through media monitoring and the ECOWARN Reporter, whose indicators captured region-specific conflict drivers and initially comprised approximately 30 reporters, including both government and civil society representatives. Following the 2017 reform, the reporter network relied entirely upon 92 non-state actors; 77 reporters from five research institutions that specialised in an analytical domain in each country – with an extra two in Nigeria – and 15 news mangers at the national WANEP offices who entered information from WARN and media sources into the ECOWARN Reporter. WARN counted 350 community monitors from over 750 WANEP-affiliated local CSOs. While news managers verified the information, regional analysts at WANEP’s headquarters checked the quality of national reports for the ECOWARN Reporter.^83^

ECOWARN’s blueprint envisaged that NERCs would rely on reports from the Early Warning Directorate to coordinate responses.^84^ This was the case with Côte d'Ivoire and with Guinea Bissau’s barely functional NERC.^85^ Liberia’s and Ghana’s NERC directors, however, deemed government sources better and faster. As these NERCs, which were staffed with civil servants and military personnel, could access potentially sensitive national intelligence, information-sharing with ECOWAS and CSOs proved problematic. Liberia’s NERC, moreover, leveraged the National Peacebuilding Office’s network of 715 community monitors.^86^

WANEP representatives saw the added value of their data in the ability to register low-intensity local conflicts and issues government reporters would sweep under the carpet. Crucially, the foremost advantage of the partnership model was that WANEP did not merely feed information into ECOWARN but could utilise its WARN data for independent analyses and civil society responses. WARN moreover comprised additional country-specific indicators to gather granular data on human security concerns for independent WANEP reports.^87^ Maintaining the independent CEWRS was challenging, however, as WANEP grappled with a high turnover of volunteer monitors and the cost of training, equipment and transport. Monitors’ fatigue with repetitive tasks impaired the data’s consistency. Repeated ECOWARN reforms and novel conflict risks required costly adjustments and complicated the collection of longitudinal data.^88^

WANEP practised a strict policy of political neutrality to gain ECOWAS’ and governments’ trust.^89^ Integrating CSOs into NERCs, however, involved difficulties, as their directors raised concerns over the sustainability, capacity and politics of CSOs, which may tweak data to serve their own rather than state interests.^90^ Conversely, civil society actors questioned governments’ commitment to sustaining NERCs and public employees’ early warning expertise and loyalties.^91^

Overall, the integration of CSOs enabled the collection of local-level data on a considerable scale thanks to WANEP’s comparatively dense national monitoring networks. Crucially, since WANEP maintained a free-standing CERWS to feed into ECOWARN, it could use its data independently for analyses and responses. While ECOWARN entirely relied on non-state reporters, several NERCs reverted to state sources and intelligence.

### ECOWARN analysis

In the ECOWAS Early Warning Division, a WANEP Liaison Officer was embedded into the team of five ECOWARN analysts. Besides coordinating WANEP with ECOWAS structures, the officer used WARN to cross-check findings, provided analyses on demand, proposed responses, and added country-specific information.^92^ WARN analyses were produced by national news mangers and verified by WANEP’s regional cluster analysts.^93^

In Burkina Faso, Côte d’Ivoire, Guinea and Liberia, the national coordinators of WANEP and other NGOs participated in weekly NERC meetings to discuss responses. However, since NERCs used confidential government information, CSOs were not involved in analyses.^94^ While ECOWARN fully integrated WANEP at the centre and NERCs were meant to boost civil society participation in term of the reformed ECOWARN blueprint, in practice, this study suggests that the introduction of NERCs, thus, diminished civil society’s role in analyses.^95^

### ECOWARN warning and response

ECOWARN was designed to warn regional and national decision-makers. The ECOWARN Directorate issued confidential reports to ECOWAS’ Vice President and President, shared selected information horizontally with ECOWAS departments, issued briefings for external partners including embassies and the AU, and generated public briefs.^96^ Interviewees cited several reasons for a warning-response gap. These included communicating customised warnings to different recipients, convincing decision-makers of their validity and urgency, and making actionable recommendations in succinct briefs. The lack of a clear policy on whether ECOWAS or states should respond, in addition to bureaucratic inertia and the tedious communication of warnings between regional and national actors, delayed responses. Finally, it was observed that governments prevented the ECOWAS Commission, whose members they had nominated, from responding to intrastate conflicts.^97^

Remarkably, WANEP was permitted to inform ECOWAS decision-makers based on WARN reports in quarterly briefings to ECOWAS ambassadors and in meetings with the vice president. The system, however, lacked feedback mechanisms to evaluate if decision-makers considered reports expedient; WANEP representatives observed that warnings and recommendations were often ignored as governments’ interests took priority.^98^

ECOWARN and response mechanisms for preventive diplomacy and mediation by ECOWAS were not formally integrated. ECOWARN, however, shared information with the Mediation Facilitation Division, which arranged stakeholder consultations for ECOWAS mediators and partnered with WANEP for training.^99^ WANEP likewise supported the reconstitution of the Council of the Wise, which was defunct between 2016 and 2020.^100^ ECOWARN analyses were not systematically leveraged for policymaking across ECOWAS departments but informed policies on national peace infrastructures. An ECOWAS policy on presidential term limits which WANEP helped to draft to prevent coups was not adopted. The ECOWAS-sponsored West African Civil Society Forum (WACSOF) nominally served as the main interface to inform policymaking but lacked peacebuilding expertise.^101^ The ECPF Secretariat and Human Security & Civil Society Division offered further access points to inform ECOWAS policies.^102^

NERCs issued reports on analytic domains to government departments but restricted information-sharing with both ECOWAS and CSOs when using sensitive information. WANEP’s national offices, meanwhile, disseminated WARN reports among government and non-state actors. NERCs sometimes requested CSOs to communicate findings that criticised the government.^103^

While NERCs were introduced to improve the coordination and speed of national responses, in 2023 the seven operational centres were still developing response mechanisms. Coups d’états in Burkina Faso, Guinea, Mali and Niger dramatically disrupted the respective NERCs’ capacitation.^104^ Governments’ commitment to maintaining costly NERCs varied sharply; for instance it was absent in Guinea Bissau, while Liberia greatly valued conflict prevention. ECOWAS did not require NERCs to involve CSOs but mandated WACSOF to evaluate national responses. NERC directors regarded ministries rather than CSOs as main responders, but aimed to coordinate state and non-state actors.^105^ In Ghana and Liberia, peacebuilding NGOs could undertake independent preventive and dialogue initiatives to mitigate electoral and inter-communal conflicts. In several countries, however, CSOs lacked the freedom and capacity for such interventions.^106^

In sum, ECOWAS, permitted WANEP to warn decision-makers and offered channels to CSOs to inform mediations and policymaking. Contrary to expectation, NERCs provided little room for participation in warnings and responses. Thanks to WARN, WANEP-affiliates could nevertheless warn stakeholders and undertake preventive interventions where governments provided civic space.

## IGAD’s conflict early warning and response mechanism^107^

IGAD played a pioneering role when adopting the 2002 Protocol on the Establishment of a Conflict Early Warning and Response Mechanism.^108^ The decentralised CEWRS was designed to enable government-led national and local-level responses and became operational in 2003. For over a decade, CEWARN focused on pastoralist conflicts in three cross-border clusters between Ethiopia, Djibouti, Somalia, Kenya, Uganda and South Sudan. While such cross-border conflicts required intergovernmental coordination, intrastate conflicts fell outside CEWARN’s mandate. To prevent pastoralist conflicts, CEWARN built a network of field monitors and local peace committees in the Dikhil, Karamoja and Somali clusters.

**Figure 3.**
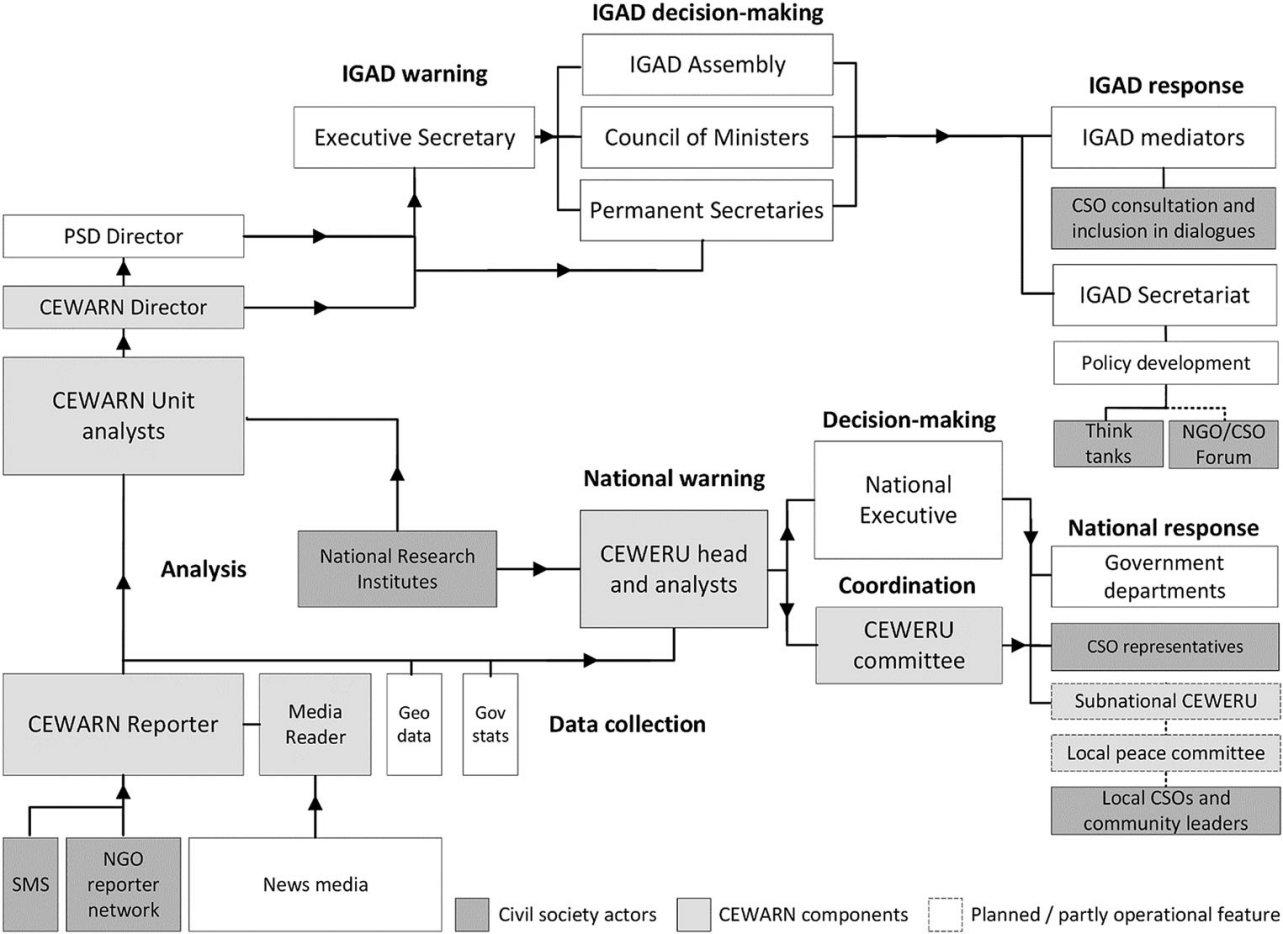
CSOs in IGAD’s early-warning-response process. *Source:* The author created this organigram based on the interviews and documents cited in the IGAD case study.

The 2012 CEWARN Strategy Framework fundamentally transformed the system to monitor a range of conflict vulnerabilities relating to the economy, governance, social affairs, security and environment across all IGAD countries. The central CEWARN Unit coordinated data collection, analyses, information-exchange and regional responses. National Conflict Early Warning and Response Units (CEWERUs), based in national ministries, oversaw all operations and orchestrated national responses. National research institutes (NRIs) and CEWERUs were responsible for national-level analyses. Following the 2012 strategy change, CEWARN introduced media monitoring and expanded its reporter networks beyond cross-border clusters (Figure 3).^109^

### IGAD policy framework

IGAD policies promoted CSO participation in CEWARN but left modalities at the discretion of governments, which are able to tightly regulate CSOs’ selection, freedoms and contributions. The CEWARN Protocol envisaged that CEWERU Committees would include non-state actors, whereby NGOs would assist with data collection and research institutions analyses. CEWERUs should make findings available to civil society ‘to the greatest extent possible’ but states may restrict information for national security reasons.^110^ The 2012 Strategy Framework altered CSOs’ roles by dramatically expanding CEWARN’s geographic and analytic range. Underscoring the involvement of private sector and civil society entities as a key strength, it aimed to build implementation capabilities among national actors in CEWERUs to promote ownership and human security.^111^

Researchers greeted the framework with scepticism, given the lack of resources and the record of governments’ unwillingness to heed warnings on intrastate conflicts.^112^ In addition, although the strategy exceeded CEWARN’s Protocol, the latter was not amended, thus leaving the decision to involve CSOs to states.^113^ IGAD’s regional and gender strategies likewise advocated CSO participation in prevention, but a Protocol on Preventive Diplomacy and Mediation that would leverage CEWARN and promote inclusion in peacemaking was not ratified.^114^

### CEWARN operationalisation

International NGOs’ contribution to CEWARN’s operationalisation and local civic actors’ vital role in cross-border clusters were documented by involved experts and a CEWARN-published compendium.^115^ The CEWARN Reporter was, however, designed by a commercial software developer, Virtual Research Associates. By collecting data through community monitors, CEWARN developed predictive capacity for incidents including cattle raids. In response, CEWERUs alerted community leaders and police, while local peace committees devised preventive action.^116^ Research on clusters highlighted problems relating to community monitors’ biases, training, information overload, communication infrastructure, distrust by communities, and harassment by local officials.^117^ While CEWARN’s decentralised design should enable participatory security governance, Kasaija found that by 2013, only three countries had functional CEWERUs.^118^ The negotiated integration of CEWERUs into ministries and states’ reluctance to capacitate an intergovernmental information-gathering system with non-state actors in sensitive borderlands greatly delayed the operationalisation.^119^ Some governments merely appointed CEWERU heads without inclusive committees.^120^ Participating CSOs were disgruntled by their lack of access to collected data and the absence of their concerns from CEWARN publications.^121^ In sum, the original CEWARN could prompt civic responses in clusters, but without functional participatory CEWERUs, it lacked effective links to response mechanisms in most countries.^122^

### CEWARN data collection

The CEWARN Strategy Framework was implemented in 2013, but as gathering information on 26 conflict types across five analytic domains bore technical and financial difficulties, data collection started only in 2016. The strategy required enhanced collection methods and partnerships. As media monitoring became the primary method, the merits of a resource-intensive reporter network to gather data on a broad spectrum of subjects across countries were weighed against the cost. IGAD decided to retain its CEWARN Reporter platform but altered the algorithm and questionnaire. It replaced the individual community monitors in the clusters with up to 60 reporters per country recruited from established NGOs and universities.

The decision to retain a modified network of civil society reporters was informed by CEWARN’s aim to ‘democratise security’, foster public ownership, and avoid duplicating intelligence. Expanding the original net of community monitors, who had become subject to violent attack and whose reach was confined to their surroundings, was unattainable. Therefore, CEWARN banked on better-qualified reporters in NGO and universities, hoping to leverage their wider knowledge, personal networks, and ability to discern relevant information and counter media bias.^123^ NGOs hoped, moreover, to be able to raise donor-funding independently since CEWARN could not centrally finance the extensive network.^124^ CEWARN shortlisted NGOs for the network based on their specialisation in analytic domains and governments’ endorsement, which was essential to ensure states trusted CEWARN reports.^125^ As a result, NGO and IGAD representatives suggest, the selection process often favoured docile NGOs, some of which fell short of technical requirements, while excluding better-capacitated outspoken organisations. In countries with highly restricted civic freedoms, the vetting process likely exacerbated self-censorship to the detriment of reporting and analyses.^126^

Up until 2021, CEWARN was collecting data in all domains through approximately 60 reporters in seven IGAD countries. As NGO reporters were urban-based, networks were thin in rural areas. The reporters could not, however, complement media-based data with information on local-level dynamics on a meaningful scale. Their situation and incident reports were valuable at first, but the data deteriorated gradually as fatigued reporters entered inconsistent information. Eventually, the envisaged model proved unsustainable, for NGOs expected full compensation for work-intensive tasks, while CEWARN provided only training and equipment as it could not acquire required donor funds centrally. In 2023, CEWARN was, therefore, experimenting with adjustments while holding on to civil society reporters who had been integral to CEWARN since its inception. The likelihood is that, in the future, NGO reporters will primarily verify and evaluate findings rather than seek to complement media-based data.^127^

### CEWARN analysis

The CEWARN Unit’s country experts and CEWERU’s analysts were responsible for data verification, triangulation and analysis. Country-specific information was restricted to the CEWERU, data managers, national analysts and CEWARN directorate. Neither the CEWARN Unit nor CEWERUs included NGO analysts, but non-state actors were involved through NRIs and, in rare cases, CEWERU Committees.^128^ The 2012 framework increased the number of NRIs that specialised in analytic domains from one to five per country. The 45 NRIs mostly comprised universities that were selected owing to their sectoral expertise and governments’ approval, and with an aim to tap into their ongoing research. Besides quarterly sectoral situation reports, NRIs were expected to provide in-depth briefings on demand. Ethiopia’s initial NRI also developed response options.^129^ The 45 NRIs considerably boosted CEWARN's analytical capacity according to its director, and their analyses were valued in the planning of responses by CEWERUs, whose committees include NRIs.^130^ However, the research partnerships proved challenging, as scarcely-resourced NRIs could not complete periodic nationwide assessment studies. NRIs that were previously compensated by CEWARN now had to finance services through government and donor contributions. In addition, though CEWERUs should, in principle, give NRIs access to selected CEWARN data,^131^ in practice information-sharing was heavily restricted as CEWARN had to treat data confidentially to maintain governments’ trust, or risked a backlash from state security agencies. NRIs, therefore, served to triangulate rather than do analyses with CEWARN data.^132^ Researchers had to exercise caution and self-censorship when analysing governance-related vulnerabilities in countries that restricted academic freedom.^133^ Without feedback mechanisms, researchers could not tell whether analyses were appropriate for decision-making and responses.^134^ In Kenya, whose peace infrastructure had inspired CEWARN, CSOs could contribute to analyses in the CEWERU Committee.^135^ Such involvement was impossible in some countries however, including in Ethiopia where the government sharply restricted access to potentially security-relevant information.^136^

### CEWARN warning and response

In the original clusters, CEWARN’s built-in response mechanisms were the outstanding feature and primary activity of the decentralised system. Besides prompting interventions by police and community leaders, CEWARN drafted and accompanied the implementation of prevention policies on cross-border trade and livestock markets to avert rustling. The framework shifted focus from local response to developing early warning capacity nationally and regionally, and it became evident that CEWARN’s aspiration to recommend responses to vulnerabilities in all domains and states greatly exceeded its capabilities. By 2023, CEWARN had come to function as an early warning system, but is aiming to reinvigorate its response component by building capacity for structural prevention and policymaking.^137^

CEWARN was designed to enable government responses rather than to take action, but the CEWARN Unit did inform IGAD decision-makers, and thus may be credited with prompting preventive diplomacy, mediation and policy-making at the IGAD Secretariat. CEWARN reports were evaluated by its director and submitted to the Peace & Security Division Director and IGAD Executive Secretary, who is able to inform permanent secretaries, the Council of Ministers and the Assembly of Heads of States. CEWARN's decentralised design created the propensity for a warning-response gap, however, as states were the responders. Communicating warnings to governments for policy-development was especially difficult. Insiders suggested that whether warnings were passed on to IGAD and national decision-makers hinged on the personalities and nationalities of CEWARN’s director, who traditionally hailed from Kenya, and IGAD’s Executive Secretary.^138^

Although CEWARN and IGAD mediation structures lack formal links, CEWARN has produced analyses for mediations on demand and seconded staff to provide analytical support to the office of the Special Envoy for South Sudan.^139^ Whereas the MSU partnered with NGOs for knowledge-management and the mediation roster featured civic actors, IGAD never deployed these structures for mediations.^140^ The impact of inclusion mechanisms on negotiation outcomes in the IGAD-mediated South Sudan peace process has been marginal.^141^

The 2012 Strategy Framework envisaged a shift from issuing ‘fire alerts’ to long-term forecasts to tackle structural vulnerabilities through policy-development. The CEWARN Unit, therefore, produced national conflict profiles and scenarios, flagged conflict drivers for governments, and proposed policies.^142^ While country analyses remained confidential, CEWARN released regional reports that named grievances (ie, electoral mismanagement) but no culprits.^143^ The implementation of preventive policies hinged on the communication of abstract relations between structural vulnerabilities and violence, and national legislative and bureaucratic processes.^144^

In national responses, the CEWERU Committees’ multi-agency model should ensure coordination among government and non-state actors in planning and implementation. Committees were operational in Kenya and Uganda but idle in countries including Ethiopia due to government restructuring and intrastate crises. CEWARN aimed to revitalise CEWERUs, advocating compulsory reporting on committees and responses. Envisaged sub-national CEWERUs and peace committees did not materialise, however, except for Kenya’s pre-existing local committees.^145^ Hence, whereas civil society had been integral to responses in clusters, it remained marginal under the 2012 Strategy Framework.

## Conclusion: Key findings and synthesis

The discussion of how civil society participated in the early warning and response processes of the AU, ECOWAS and IGAD has served to critically assess whether participation could fulfil expectations raised in literature, policies and by proponents. Besides synthesising the findings of the case studies, this section assesses the comparative advantages of the participation models (see Table 1).

**Table 1.** CSOs in the early-warning-response process.

	AU	ECOWAS	IGAD
**Data collection**	Africa Reporter: WANEP, SAPPC, ECONET, ‘COPAC’ reporters *WANEP WARN monitors*	ECOWAS Reporter: WANEP & academic reporters *WANEP WARN monitors*	CEWARN Reporter: NGO & academic reporters Field monitor SMS
**Analysis**	WANEP Liaison Officer Ad-hoc think tank-inputs	WANEP Liaison Officer Ad-hoc think tank-inputs *WANEP WARN analyses*	National Research Institutes: quarterly reports
**Warning**	Occasional written and in-session briefings to PSC	WANEP-briefings to ambassadors (quarterly) and Vice President (occasional) ‘Briefing of NGOs by NERCs’ *WANEP WARN reports*	‘Briefing of NGOs in CEWERU Committees’
**Response**	Consultation in preventive and mediation missions Representation in PoW, FemWise, Standby Force ECOSOCC and think tank input in policy-development.	Consultation in preventive and mediation missions Representation in CoW, FemWise, Standby Force WACSOF and think tank-input in policy-development ‘Mobilisation for NERC responses’ *Responses by WANEP affiliates*	Consultation in preventive and mediation missions Mobilisation for CEWERU-coordinated responses and local peace committees NGO/CSO Forum and think tank input for policy-making

*Source:* Interviews with Clarke, Diallo, Edopu, Mensah, Merdiemah, Moyo, Okechukwu, Omogo, Respondents 11, 12, 14.

*Notes:* Those items in this table in italics are ***Independent CSO structures and activities.*** Those items in inverted commas are '**Partly operational or planned features'**.

The cases show that CSO participation could, in principle, improve data collection, analyses, warnings and responses, but in the cases under scrutiny it hardly lived up to expectations in practice. The reasons included NGOs’ low capacity, thin monitoring networks, and a lack of civic space to engage in conflict prevention in authoritarian and insecure settings. African IGOs recruited CSOs to collect information but provided little room to substantively inform analyses, warnings and responses as (inter)governmental officials committed to participation in policies but remained suspicious of non-state actors’ involvement in security matters.^146^

All three systems under study included civil society reporters for data collection, but outside West Africa, monitoring networks were too thin to meaningfully complement media-based data with information on local-level dynamics.^147^ Isolated reporters who depended on media sources could not reliably identify misinformation.^148^ Analyses were assisted by research institutions, but reports to decision-makers left minimal room for alternative perspectives and recommendations.^149^ Whereas early warning and mediation support were not integrated in all three IGOs, civic stakeholders were commonly consulted in preventive and mediation missions.^150^ NGOs’ contribution to developing preventive policies was hampered by poorly functioning IGO-CSO interfaces.^151^ National warning and response centres, where operational, were called on to coordinate government and non-state actors, but in practise were found to securitise early warning and focused on state responses.^152^ The IGOs were, thus, a long way from actualising their own participatory conflict prevention policies.^153^

By interpreting these case study findings and juxtaposing the CSO participation models of the AU, ECOWAS and IGAD with regard to conflict and early warning and response systems, this study concludes that for participation to live up to expectations, regional blocks would need to emulate the ECOWARN-WANEP model. Analogous to the West African model, NGO networks must be able to build independent CEWRS, in which civil society actors complete the basic steps of the warning-response process in parallel with (inter)governmental systems – rather than solely feed selective information at individual stages into those systems. The findings show that Eastern and Southern African NGOs that reported to the CEWS and CEWARN were denied access to aggregate data and analytical outputs to inform civic responses.^154^ Thanks to its free-standing CEWR, WANEP could, in contrast, gather deviant data and conduct independent analyses; warn ECOWAS, government and civic stakeholders based on these alternative findings; and devise local responses by WANEP affiliates.^155^ Building NGO-managed CEWRS that run in parallel to (inter)governmental systems is, thus, key to achieve that participation; only then can they meaningfully complement data with information on human security concerns which state actors may ignore, enrich analyses with deviant perspectives based on alternate data, and enable timely local responses.

## Notes

1 Interview with Respondent 31, AU, 14.05.24, Addis Ababa.

2 David Nyheim, *Early Warning and Response to Violent Conflict* (London: Saferworld, 2015), 6.

3 David Nyheim, *Preventing Violence, War and State Collapse* (Paris: OECD, 2009), 15.

4 Robert Muggah and Mark Whitlock, ‘Reflections on the Evolution of Conflict Early Warning,’ *Stability: International Journal of Security and Development* 10, no. 1 (2022): 4.

5 Nyheim, ‘Early Warning,’ 3.

6 Muggah and Whitlock, ‘Reflections’.

7 Thomas Chadefaux, ‘Conflict Forecasting and Its Limits,’ *Data Science* 1, no. 1–2 (2017): 7; Issaka Souaré and Paul-Simon Handy, ‘The State of Conflict Early Warning in Africa,’ *African Security Review* 22 (2013): 19.

8 Muggah and Whitlock, ‘Reflections,’ 4.

9 Alexander George and Jane Holl, ‘The Warning-Response Problem and Missed Opportunities in Preventive Diplomacy’ (New York: Carnegie Commission on Preventing Deadly Conflict, 1997); Christoph Meyer, Chiara De Franco, and Florian Otto, *Warning about War* (Cambridge: Cambridge University Press, 2019), 5.

10 Ulf Engel, ‘Early Warning and Conflict Prevention,’ in *The Routledge Handbook of Transregional Studies,* ed. M. Midell (London: Routledge, 2018).

11 AU, ‘Protocol Relating to the Establishment of the Peace and Security Council,’ Addis Ababa, 2002, Art 12; Interview with Respondent 11, AU, 27.09.2021, Online.

12 Ulf Engel, ‘The African Union’s Early Warning System: An Easy Come, Easy Go Regionalism?,’ Working Paper (Leipzig: Collaborative Research Centre, 2022), 11.

13 Doug Bond, ‘Constructing the CEWARN Model,’ in *Conflict Early Warning in the Horn*, ed. Paul Goldsmith (Djibouti: IGAD, 2020), 91. Interview with Respondent 14, IGAD, 20.10.2021, Online.

14 Amandine Gnanguênon, ‘Pivoting to African Conflict Prevention? (Brussels: European Union Institute for Security Studies, 2021), 4; Interviews with Camlus Omogo, IGAD, 9.08.2023, Addis Ababa; Respondent 11.

15 Jakkie Cilliers, ‘Towards a Continental Early Warning System for Africa’ (Pretoria: ISS, 2005); Engel, ‘Early Warning’; Laurie Nathan, Michelle Ndiaye, and Yahia Zoubir, ‘APSA Assessment Study’ (Addis Ababa: AU, 2015), 67–72; Alexander Noyes and Janette Yarwood, ‘The AU Continental Early Warning System,’ *International Peacekeeping* 20, no. 3 (June 2013): 249–62.

16 Gnanguênon, ‘Pivoting, 5; Laurie Nathan, ‘Africa’s Early Warning System: An Emperor without Clothes?,’ *South African Journal of International Affairs* 14, no. 1 (2007): 54; Herbert Wulf and Tobias Debiel, ‘Systemic Disconnects: Why Regional Organizations Fail to Use Early Warning Mechanisms,’ *Global Governance* 16, no. 4 (2010): 542.

17 Budapest Centre for Mass Attrocities Prevention, ‘African Regional Communities and the Prevention of Mass Attrocities’ (Budapest: BCMAP, 2016), viii; Phillip Apuuli Kasaija, ‘The Intergovernmental Authority on Development’s Conflict Early Warning and Response Mechanism,’ *African Security Review* 22, no. 2 (2013): 12; ISS, ‘Denialism Plagues Africa’s Early Warning System,’ PSC Report (Pretoria: ISS, April 2017).

18 Sara Hellmüller, ‘The Changing Role of Civil Society Actors in Peacemaking and Peacebuilding,’ in *Routledge Handbook of Peace, Security and Development*, ed. Osler Hampson et al., (Oxon: Routledge, 2020).

19 Michael Edwards, ‘Introduction,’ in *The Oxford Handbook of Civil Society*, ed. Michael Edwards (New York: Oxford University Press, 2011), 3.

20 Hellmüller, ‘The Changing Role’.

21 Chukwuemeka Eze and Osei Frimpong Baffour, ‘Contributions of Early Warning to the African Peace & Security Architecture,’ in *The State of Peacebuilding in Africa*, ed. Terence McNamee and Monde Muyangwa (Cham: Springer, 2021), 182.

22 Godfrey Musila, ‘Early Warning and the Role of New Technologies in Kenya,’ in *New Technology and the Prevention of Violence and Conflict*, ed. Francesco Mancini, (New York: International Peace Institute, 2013), 42–55; John Mark Opoku, ‘West African Conflict Early Warning and Early Response System’ (Accra: KAIPTC, 2007), 6.

23 Cilliers, ‘Towards,’ 7; Heinz Krummenacher et al., ‘Practical Challenges in Predicting Violent Conflict’ (Bern: Swisspeace, 2001), 4.

24 Amandine Gnanguênon, ‘Afrique de l’Ouest : faire de la prévention des conflits la règle’ (Bruxelles: Observatoire Boutros-Ghali, 2018); WANEP, ‘Practice Guide on CSOs Engagement with Intergovernmental Organizations’ (Accra: WANEP, 2019).

25 Michael Aeby and Jamie Pring, ‘The Buzz about Inclusion in Peace Research, Policy and Practice in IGAD and SADC,’ in *APSA Inside-Out*, ed. Ulf Engel et al. (Leiden: Brill Publishers, 2021), 186.

26 Interview with Respondent 11.

27 IGAD, ‘CEWARN Strategy Framework 2012–2019,’ Djibouti, 2012.

28 Interview with Ifeanyii Okechukwu, WANEP, 13.09.2021, Accra.

29 Michael Aeby and Jamie Pring, ‘Development Trajectories of Mediation Support Structures in the AU, ECOWAS, IGAD and SADC,’ *South African Journal of International Affairs* 30, no. 1 (2023), 1–24.

30 Engel, ‘Early Warning and Conflict Prevention’.

31 Interviews with Habib Kambanga, SADC, 10.06.2021,

32 Interviews with Tamrat Kebede, Inter-Africa Group, 16.06.2023, Addis Ababa; Respondent 14; Respondent 20, ECONET, 18.10.2021; Respondent 21, ECONET, 18.10.2021, Online, Respondent 22, SAPPC, 19.10.2021, Online; Respondent 23, SAPPC, 18.10.2021, Online; Respondent 30, LPI, 15.06.2023, Addis Ababa.

33 Interviews with Alimou Diallo, USAID, 21.09.2021, Online; Peter Edopu, ECONET, 14.10.2021, Online; Osei Baffour Frimpong, WANEP, 9.10.2021, Online; Edwige Mensah, WANEP, 18.10.2021, Online; Nqobile Moyo, SAPPC, 10.04.2021, Online; Omogo; Respondent 12, IGAD, 11.05.2021; Respondents 12, 21.

34 Interviews with Edopu; Moyo; Respondent 29, IGAD, 14.06.2023, Addis Ababa; Respondents 11, 14.

35 Interviews with Francis Acquah-Aikins, WANEP, 12.06.2019, Abuja; Ronald Clarke, NERC Liberia, 10.07.2021; Gabriel Ekanza, NERC Côte d'Ivoire, 10.12.2021, Online; Constant Gnancadja, ECOWAS, 12.12.2019, Abuja; Phillip Kollie, WANEP, 10.5.2021. Online; Brown Odigie, ECOWAS, 12.03.2019, Abuja; Okechukwu; Ansoumane Souaré, WANEP, 21.06.2023 Online.

36 AU, ‘Protocol’ Art 12; Interview with Respondents 11, 31.

37 Ulf Engel, ‘The African Union’s Conflict Early Warning System Is No More,’ *The Conversation*, 24.05.2022, http://theconversation.com/the-african-unions-conflict-early-warning-system-is-no-more-what-now-183469; Interviews with Respondent 30, ISS, 15.06.2023, Addis Ababa; Respondent 11, 31.

38 AU, ‘The CEWS Handbook’ (Addis Ababa: AU, 2008), 18, 35, 72.

39 AU, ‘Livingstone Formula: Conclusions on a Mechanism for Interaction between the PSC and Civil Society, Addis Ababa, 2008; AU, ‘Maseru Conclusions on Enhancing the Implementation of the Livingstone Formula, Addis Ababa, 2014.

40 Interviews with Désiré Assogbavi, Oxfam, 2.2.2020, Online; Respondent 28, IPSS, 3.7.2023, Online; Respondent 11.

41 Interviews with Respondent 5, ISS, 31.01.2020, Online; Respondent 11; John Pokoo, KAIPTC, 17.9.2021, Accra.

42 Cilliers, ‘Towards,’ 6.

43 Ulf Engel, ‘Knowledge Production on Conflict Early Warning at the African Union,’ *South African Journal of International Affairs* 25, no. 1 (2018): 576.

44 Nathan, Ndiaye, and Zoubir, ‘APSA,’ 67; ISS, ‘Denialism’; Interviews with Respondent 4, ISS. 23.1.2020, Addis Ababa; Respondent 28.

45 Engel, ‘Knowledge,’ 122; Engel, ‘The African Union,’ 8; Gnanguênon, ‘Pivoting,’ 6; Sue Mbaya, ‘African Union–Civil Society Relations’ (Pretoria: ISS, 2023), 30; Interview with Respondent 2, AU, 28.01.2020, Addis Ababa.

46 Interview with Respondent 11.

47 Engel, ‘Knowledge’; Interview with Respondent 11.

48 Interviews with Mensah, Respondents 11, 31.

49 Interviews with Mensah, Respondents 11, 31.

50 Interviews with Liphapang Monesa, SAPPC, 18.10.2021, Online; Moyo, Respondent 22.

51 Interviews with Edopu, Respondents 11, 20, 21

52 Interview with Respondent 31.

53 Interviews with Frimpong, Mensah, Respondent 11.

54 Interviews with Mensah, Monesa, Moyo, Respondents 20, 21, 22, 23.

55 Interviews with Mensah, Monesa, Moyo, Respondents 20, 21, 22, 23.

56 Engel, ‘Knowledge,’ 123.

57 Interview with Respondent 11.

58 Interviews with Mensah, Respondent 23.

59 Engel, ‘The African Union,’ 202; Interviews with Respondents 28, 30, 31.

60 Interviews with Respondents 5, 11, 30.

61 Interviews with Respondents 5, 28.

62 Interviews with Frimpong, Mensah, Respondents 11, 28.

63 Interviews withRespondents 28, 31.

64 AU, ‘Protocol,’ Art 12; Interview with Respondent 11.

65 Interviews with Simon Badza, AU, 25.01.2020, Addis Ababa; Respondent 11.

66 Interviews with Assogbavi; Brenda Mofyia, AU, 22.01.2020, Addis Ababa; Respondent 23.

67 AU, ‘Protocol’; Interview with Respondent 11.

68 Interview with Respondent 3, AU, 23.01.2020, Addis Ababa.

69 Interviews with Stella Sabiiti, AU, 21.1.2020, Addis Ababa; Respondent 28.

70 Interviews with Michelle Ndiaye-Ntab, IPSS, 31.01.2020, Addis Ababa; Respondent 1, AU, 28.01.2020, Addis Ababa.

71 Interviews with Assogbavi, Yesmirach Kebede, LPI, 23.01.2020, Addis Ababa; Jean Mfasoni, AU, 21.01.2020, Addis Ababa; Mofyia; Respondent 11.

72 Michael Aeby, ‘Civil Society Participation in Peacemaking and Mediation Support in the APSA, (Cape Town: Institute for Justice and Reconciliation, 2021), 1–7; Mbaya, ‘African Union,’ iv–vi; Mbaya, ‘African Union,’ iv–vi.

73 ECOWAS, ‘Protocol Relating to the Mechanism for Conflict Prevention, Management, Resolution, Peace-Keeping and Security,’ Abuja, 1999 Art IV.

74 WANEP, ‘Practice Guide,’ 31.

75 Interviews with Diallo; Joseph Merediemah, NERC Ghana, 23.09.2021, Accra; Ansoumane Souaré, WANEP, 15.09.2021 Accra.

76 ECOWAS, ‘ECOWAS Conflict Prevention Framework (ECPF),’ Abuja, 2008, 69–74; Interview with Odigie.

77 Diallo, interview, 21 September 2021; ECOWAS, ‘ECPF,’ 16–18.

78 ECOWAS, ‘ECOWAS Mediation Guidelines,’ Abuja, 2018, 55.

79 Acquah-Aikins, interview; Mensah, Interview; WANEP, ‘Practice Guide,’ 25.

80 Gnanguênon, ‘Afrique,’ 15; Opoku, ‘West African,’ 6.

81 Eze and Frimpong, ‘Contributions,’ 182.

82 Gnanguênon, ‘Afrique’; Interview with Amandine Gnanguênon, UNU-CRIS, 22.01.2020. Email.

83 Interviews with Kollie, Okechukwu, Souaré, 21.06.2023.

84 Interview with Diallo.

85 Interviews with Ioba Embalo, NERC Guinea Bissau, 10.07.202, online; Ekanza.

86 Interviews with Clarke; Linda Darkwa, LEDRIZ, 22.09.2021, Online; Merediemah; Souaré, 21.07.2023.

87 Interviews with Acquah-Aikins, Diallo, Frimpong, Kollie, Mensah, Souaré, 15.09.2021.

88 Interview with Okechukwu.

89 Interview with Okechukwu.

90 Interviews with Clarke, Embalo, Merediemah, Ekanza.

91 Interviews with Diallo, Frimpong, Souaré 15.09.2021.

92 Interviews with Acquah-Aikins, Diallo, Mensah.

93 Interviews with Kollie, Okechukwu, Souaré 15.09.2021.

94 Interviews with Clarke, Darkwa, Embalo, Mensah, Merediemah, Ekanza.

95 Interviews with Clarke, Diallo, Frimpong, Merediemah, Ekanza, Souaré 15.9.2021.

96 Interviews with Acquah-Aikins, Mensah, Souaré 15.09.2021.

97 Interviews with Emma Birikorang, KAIPTC, 22.09.2021, Accra; Clarke; Frimpong; Mensah; Pokoo, KAIPTC, Souaré, 15.09.2021.

98 Interviews with Acquah-Aikins; Frimpong; Okechukwu; Souaré 15.09.2021.

99 Interviews with Levinia Addae-Mensah, WANEP, 2.04.2020, Diallo, Odigie.

100 Interviews with Diallo, Gnancadja.

101 Interviews with Diallo, Komlan Messie, WACSOF, 12.10.2019, Abuja.

102 Interviews with Gnancadja, Raheemat Momodu, ECOWAS, 12.12.2019, Abuja.

103 Interviews with Clarke, Ekanza, Embalo, Kollie, Merediemah.

104 Interviews with Diallo, Souaré 21.06.2023.

105 Interviews with Clarke, Embalo, Ekanza.

106 Interviews with Diallo, Frimpong, Kollie, Okechukwu.

107 A variation of the case study was published as Michael Aeby, ‘CEWARN’s Strategy Framework and Civil Society Partnership,’ *Conflict Trends*, 2023, No. 2, 3–14.

108 IGAD, ‘Protocol on the Establishment of a Conflict Early Warning and Response Mechanism, Djibouti, 2002.

109 Paul Goldsmith, ‘Introduction,’ in *Conflict Early Warning in the Horn*, ed. Paul Goldsmith (Djibouti: IGAD, 2020), 8–17; interview with Respondent 12.

110 IGAD, ‘Protocol,’ Art 11–12.

111 IGAD, ‘CEWARN Strategy,’ 24–61.

112 Kasaija, ‘The Intergovernmental’; Rania Hassan, ‘CEWARN’s New Strategy Framework,’ *African Security Review* 22, no. 2 (2013): 26–38.

113 Interviews with Omogo; Respondent 12.

114 IGAD, ‘Draft Protocol on Preventive Diplomacy and Mediation,’ Djibouti, 2019.

115 Goldsmith, ‘Introduction’; Cirû Mwaûra and Susanne Schmeidl, eds., ‘Early Warning and Conflict Management in the Horn of Africa’ (Lawrenceville: Red Sea Press, 2002).

116 Goldsmith, ‘Introduction,’ 8–17; Interview with Respondent 12.

117 Bond, ‘Constructing the CEWARN Model,’ 92; A. K. Kaiza, ‘Mapping Conflict,’ in *Conflict Early Warning in the Horn*, ed. Paul Goldsmith (Djibouti: IGAD, 2020), 81; Helena Puig Larraui, ‘New Technologies and Conflict Prevention in Sudan and South Sudan,’ in *Early Warning and the Role of New Technologies* ed. Francesco Mancini, (New York: International Peace Institute, 2013), 87; Musila, ‘Early Warning,’ 55.

118 Kasaija, ‘The Intergovernmental,’ 12.

119 Interviews with Respondent 25, ISS, 6.9.2023, Online; Respondents 29, 30.

120 Interview with Omogo.

121 Musila, ‘Early Warning,’ 55; Larraui, ‘New Technologies,’ 85; Life & Peace Institute, ‘Civil Society and Regional Peacebuilding in the Horn’ (Addis Ababa: LPI, 2014), 25.

122 Kaiza, ‘Mapping,’ 82; Kasaija, ‘The Intergovernmental,’ 12.

123 Interviews with Omogo, Respondents 25, 29, 30.

124 Interviews with Respondent 12, 14.

125 Interviews with Omogo, Respondent 29.

126 Interviews with Kebede; Tamiru Lega, LPA, 15.6.2023, Addis Ababa; Respondents 14, 21, 25, 30.

127 Interviews with Omogo, Respondent 29.

128 Interviews with Respondent 12, 15, 29.

129 Interviews with Kebede, Respondent 25, 29.

130 Interview with Omogo.

131 Interviews with Omogo, Respondent 12, 14, 29.

132 Interviews with Respondents 25, 30; Respondent 32, IGAD, 23.05.2024, Online.

133 Interviews with Kebede, Lega.

134 Interviews with Kebede, Respondents 14, 15.

135 Interviews with Omogo, Respondents 14, 15.

136 Interviews with Kebede, Lega, Respondents 25, 30.

137 Interviews with Omogo, Respondents 25, 29, 31.

138 Interviews with Omogo, Respondents 14, 18, 25, 30.

139 Interviews with Omogo, Respondents 23, 25.

140 Interviews with Ahmed Hersi, IGAD, 2.3.2022, Online, Respondents 18, 27.

141 Interviews with Stephen Oola, IGAD, 3.10.2022, Online; Respondent 18, IGAD, 16.6.2023, Addis Ababa.

142 Interviews with Omogo, Respondents 12, 14.

143 IGAD, ‘CEWARN Regional Conflict Profiling 2021,’ Addis Ababa, 2021; IGAD, ‘CEWARN Quarterly Early Warning Report,’ Addis Ababa, January 2022.

144 Interviews with Kebede, Lega, Omogo, Respondent 15.

145 Interview with Omogo, Respondents 25, 29, 30.

146 Interviews with Diallo, Edopu, Frimpong, Mensah, Moyo, Omogo, Respondents 11, 12, 21.

147 Interviews with Edopu, Moyo, Respondent 11, 14, 29.

148 Interviews with Kebede, Respondents 14, 20, 21, 22, 23, 30.

149 Interviews with Diallo, Frimpong, Mensah, Okechukwu, Respondent 11, 12, 14, 25, 27, 28.

150 Interviews with Diallo, Hersi, Ndiaye Ntab, Odigie, Respondent 1.

151 Interviews with Assogbavi, Diallo, Kebede, Lega.

152 Interviews with Clarke, Ekanza, Embalo, Kebede, Merediemah, Okechukwu, Respondents 11, 14, 25, 28, 29.

153 AU, ‘CEWS Handbook,’ 35; ECOWAS, ‘ECPF,’ 16; IGAD, ‘CEWARN Strategy,’ 24.

154 Interviews with Edopu; Moyo; Respondent 11, 14, 29.

155 Interviews with Diallo, Okechukwu, Souaré.

